# Analyte and matrix method extension of per- and polyfluoroalkyl substances in food and feed

**DOI:** 10.1007/s00216-023-04833-1

**Published:** 2023-08-02

**Authors:** Susan Genualdi, Wendy Young, Elsie Peprah, Cynthia Srigley, Christine M. Fisher, Brian Ng, Lowri deJager

**Affiliations:** Center for Food Safety and Applied Nutrition, US Food and Drug Administration, 5001 Campus Drive, College Park, MD 20740 USA

**Keywords:** PFAS, Food, LOQ, Method development

## Abstract

The development and expansion of analytical methods for per- and polyfluoroalkyl substances (PFAS) in food are essential for the continued monitoring of the United States (US) food supply and assessments of dietary exposure. In March 2022, the European Union Reference Laboratory for Halogenated Persistent Organic Pollutants in Feed and Food (EURL POPs) released a guidance document covering priority PFAS of interest, including analytical method parameters and limits of quantification (LOQs). As a result, the Food and Drug Administration (FDA) began method extension work to incorporate ten new additional analytes to method C-010.02 including long-chain perfluorosulfonic acids, fluorotelomer sulfonates, and perfluorooctane sulfonamide. Four long-chain carboxylic acids were also validated across all foods, which were previously added to C-010.02 but only validated in seafood. In December 2022, the European Union published Commission Regulation 2022/2388, establishing maximum levels for perfluorooctane sulfonic acid (PFOS), perfluorooctanoic acid (PFOA), perfluorononanoic acid (PFNA), and perfluorohexane sulfonic acid (PFHxS) in certain foodstuffs, primarily fish, molluscs, crustaceans, and eggs. As a result, the FDA method was evaluated for performance in reaching LOQs defined in Commission Regulation (EU) 2022/1431. The FDA method was found to be able to reach all required LOQs for analytes in matrices with established maximum levels. Currently, method detection limits (MDLs), which are used by the FDA as the lower limit for reporting PFAS in surveillance samples, were in the same range as defined indicative levels. With further method modifications, required LOQs could be met in fruits, vegetables, and milk. Reaching the lower targeted LOQs for these food matrices will require moving the method to an instrument that can provide increased signal:noise gains at the lower limits of quantification.

**Graphical abstract:**

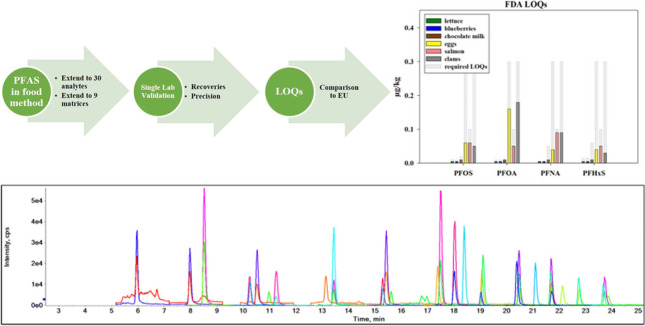

## Introduction

Per- and polyfluoroalkyl substances (PFAS) are fluorinated substances that have been in production since the 1950s [1]. They consist of carbon–fluorine bonds of varying chain lengths with hydrophilic end groups, typically carboxylic or sulfonic acids. Polyfluoroalkyl substances have one or more carbons where fluorine is substituted for hydrogen allowing the compounds to undergo degradation through the substitution of hydrogen atoms by hydroxyl radicals and with subsequent reactions (e.g., desulfonation, carboxylation, loss of CF_2_) to form stable end products [2]. Due to the high stability of these compounds, they have been used in upholstery, food contact articles, packaging coatings, carpets, and clothes as oil and water repellants. Additionally, they have been used in aqueous film-forming foams (AFFF) and the composition of these foams has changed from being largely perfluoroooctane sulfonic acid (PFOS) based to fluorotelomer-based foams in recent years [3]. PFAS have become widespread environmental contaminants due to their persistent nature and ability to bioaccumulate and uptake into plants and animals.

Exposure to humans can occur through dietary exposure from contaminated water and food, and inhalation of indoor air [4]. In order to monitor the food supply for PFAS contamination, the FDA has developed analytical methods for PFAS in a wide variety of foods. Initially, the method included 16 analytes and was used for the analysis of 93 regional Total Diet Study samples which included dairy, meats, grains, and produce [5]. This method was then revised and extended for use in 150 processed foods [6]. Recently, for a seafood survey of 81 samples, the method was extended to include 4 additional long-chain carboxylates from C11–C14, which have been previously shown to uptake into seafood [7]. This method was also expanded to silage samples and was used for the analysis of corn silage fed to cows raised on water and feed that had been contaminated by AFFF use on a nearby Air Force base [8].

In 2022, the EURL POPs released a guidance document for analytical methods for the determination of PFAS in foods outlining analytes of interest, validation parameters, and analytical method performance criteria. Due to recommendations in this document, five long-chain sulfonic acids from C9–C13, and perfluorooctane sulfonamide (FOSA), which is a precursor for PFOS, were added to the analyte list for method extension. Additionally, four fluorotelomer sulfonates (FTSs) including 4:2, 6:2, 8:2, and 10:2 were added to the analytical method due to their prevalence in AFFF. In order to continue expanding the analytical method for additional analytes and matrices, a single lab validation was performed for 30 PFAS on seven food and two feed matrices using the extraction and instrumental methods previously developed at the FDA [6, 8].

The objectives of this research were to perform an analyte and matrix extension to method C-010.02 [9] to include 30 analytes of interest and additional food and feed samples, modify previous versions of the method to enhance transferability to outside labs, and compare FDA method detection limits (MDLs) and the limits of quantification (LOQs) to those set by the EURL POPs.

## Materials and methods

### Samples

Samples used for the matrix extension include the same as previously tested in Total Diet Study samples (lettuce, milk, salmon, bread) [5, 6]. In this study, chocolate milk was chosen instead of regular milk, due to known interferences with perfluoropentanoic acid (PFPeA) in chocolate-containing foods, and eggs due to known cholic acid interferences [6]. Additional foods of interest included clams, due to the previous detection of PFOA at elevated concentrations [10], and blueberries, which are a highly pigmented food. Given the increased interest in PFAS in animal feeds and the use of this method for New Mexico silage samples [8], silage and corn obtained from our state partners in New Mexico and Maine were also validated.

### Chemicals

Analytical stock standards were purchased from Absolute Standards (Hamden, CT) in the form of a custom mix. This mix contained the following: perfluorobutanoic acid (PFBA); perfluoropentanoic acid (PFPeA); perfluorohexanoic acid (PFHxA); perfluoroheptanoic acid (PFHpA); linear and branched mixture of perfluorooctanoic acid (br-PFOA); perfluorononanoic acid (PFNA); perfluorodecanoic acid (PFDA); perfluoroundecanoic acid (PFUdA); perfluorododecanoic acid (PFDoA); perfluorotridecanoic acid (PFTrDA); perfluorotetradecanoic acid (PFTeDA); potassium perfluorobutanesulfonate (PFBS); sodium perfluoropentanesulfonate (PFPeS); linear and branched sodium perfluorohexanesulfonate (PFHxS); sodium perfluoroheptanesulfonate (PFHpS); linear and branched mixture of potassium perfluorooctanesulfonate (br-PFOS); per-fluoro2,3,3,3-tetrafluoro-2-(1,1,2,2,3,3,3-heptafluoroproproxy)propanoic acid (HFPO-DA); sodium dodecafluoro-3H-4,8-dioxanonanoate (NaDONA); potassium 9-chlorohexadecafluoro-3-oxanonane-1-sulfoate (9Cl-PF3ONS); potassium 11-chloroeicosafluoro-3-oxaudecane-1-sulfonate (11Cl-PF3OUdS), perfluorononanesulfonate (PFNS), perfluorodecanesulfonate (PFDS), perfluoroundecane sulfonate (PFUdS), perfluorododecane sulfonate (PFDoDS), perfluorotridecane sulfonate (PFTrDS), perfluorohexane sulfonic acid (4:2 FTS), perfluorooctane sulfonic acid (6:2 FTS), perfluorodecane sulfonic acid (8:2 FTS), perfluorododecane sulfonic acid (10:2 FTS), perfluorooctane sulfonamide (FOSA). This standard mix was provided in a concentration of 2 µg/mL reported as an anion concentration, so no additional salt corrections were needed. The labeled surrogate standards were purchased from Wellington Laboratories; Guelph, ON, Canada (M3PFBA, M3PFPeA, MPFHxA, M8PFOA, MPFUdA, MPFDoA, MPFTeDA, M3PFBS, MPFHxS, M8PFOS, M3HFPO, M8FOSA) in the form of a custom mix at 2 µg/mL and Cambridge Isotopes Laboratories; Tewksbury, MA (^13^C_2_d_4_ 4:2 FTS ^13^C_2_d_4_ 6:2 FTS, ^13^C_2_d_4_ 8:2 FTS ^13^C_2_d_4_ 10:2 FTS) in a custom mix at 1 µg/mL. It is challenging to accurately quantify FTSs using standards labeled with only ^13^C_2_ (as offered by Wellington) because there is a contribution to the surrogate area from the naturally occurring M + 2 isotope ^34^S (4.25%) in the native FTSs in the calibration curve [11]. Therefore, Cambridge Isotopes was chosen for the labeled FTS analytes because they offer labeled standards with two deuteriums (d_4_) in addition to ^13^C_2_. The labeled internal standard added prior to injection N-ethyl-*d*_*5*_-perfluoro-1-octane-solfonamidoacetic acid (*d*_*5*_N-EtFOSAA) was purchased from Wellington.

For this method extension, the technical PFOS and PFOA standards used in previous methods [5, 6] were replaced with synthesized branched PFOS and PFOA standards from Absolute Standards (Hamden, CT). Although the use of the technical PFOS standards is common among research labs, the purity of this standard was problematic when expanding the method to other labs with certain purity requirements. As a result, synthesized branched standards for PFOS and PFOA with purities of 99% were chosen from Absolute Standards as they most closely matched the ratio of linear and branched isomers in NIST RM 8447 and NIST RM 8446. The ratio of linear to branched isomers based on the area was 68% linear and 32% branched for the Absolute branched PFOS standard which was the most consistent to the NIST RM 8447 with 64% linear and 36% branched. All solvents (methanol, water, and acetonitrile) were LC–MS Optima grade (Fisher Scientific, Waltham, MA), and mobile phase additives (ammonium acetate and 1-methyl piperidine; 1-MP) and ammonium hydroxide, which is used for the solid phase extraction (SPE) elution solvent, were also purchased from Fisher Scientific (Waltham, MA).

### QuEChERS extraction

The extraction protocol is the same as previously described [6, 8, 10]. All food samples were homogenized using an IKA tube mill (IKA Works, Inc., Wilmington, NC). Feed samples were ground using a Robot-Coupe (Ridgeland, MS) and dry ice to a powder. Briefly, 5 g of homogenized food or 1 g of homogenized feed was added to a 50 mL centrifuge tube. A smaller sample size for feed samples was needed due to co-extractives that resulted in rapidly deteriorating chromatography with 5 g of sample. The lower sample size allowed for a more effective clean-up of the matrix. Water was added to swell the matrix (5 mL for most samples, 15 mL for dry/feed samples) and 10 mL of acetonitrile was used as the extraction solvent with the addition of 150 µL of formic acid. A salt packet was then added to each sample and hand shaken or vortexed until the salt was homogenous and no clumping was observed (6000 mg MgSO_4_ and 1500 mg NaCl (ECMSSFCS-MP, UCT, Bristol, PA). The samples were shaken and vortexed for 5 min at 1500 rpm with a pulse of 70 (Glas-Col, Terre Haute, IN) and centrifuged at 10,000 rcf for 5 min. The supernatant was transferred to the dSPE tube (900 mg MgSO_4_, 300 mg PSA, and 150 mg CGB (ECMPSCB15CT, UCT, Bristol, PA). This tube was then shaken/vortexed and centrifuged using the same conditions as before. Then, 5 mL of dSPE extract was filtered through a 0.2 µm nylon filter, and a 1 mL aliquot was saved for SPE clean-up. For dry/feed samples, the 5 mL dSPE extract was concentrated to 1 mL. The combination of primary secondary amine (PSA) and graphitized carbon black (GCB) has been long known to be superior at the removal of co-extracted matrix components in QuEChERS extractions of meat and produce [12]. Although there is the possibility of additional loss of analyte due to the presence of PSA in the dSPE step, we have found this loss to be insignificant [5]. When PSA is removed, and only GCB is used, the extracts were visually much darker especially for pigmented foods and silage samples.

### SPE clean-up

The SPE clean-up is the same as previously described [6, 10]. Briefly, 1 mL of filtered acetonitrile dSPE extract is diluted to 12 mL with water. A Strata XL-AW (200 mg, 6 mL, 100 µm, Phenomenex, Torrance, CA) cartridge was washed with 6 mL of elution solvent (0.3% w/w ammonium hydroxide in methanol) and equilibrated with 5 mL of water. The sample was loaded and the cartridge was washed with 5 mL of water. The cartridge was then eluted with 4 mL of 0.3% w/w ammonium hydroxide in methanol. The sample was concentrated to approximately 1 mL and the internal standard *d*_*5*_N-EtFOSAA was added prior to analysis. During analysis, analytical standards of taurodeoxycholic (TDCA), taurochenodeoxycholic acid (TCDCA), and tauroursodeoxycholic acid (TUDCA) were run to monitor for their presence in sample extracts. These isomers of taurodeoxycholic acids are known to interfere with the 499→80 MRM transition for PFOS in foods such as eggs, milk, liver, meat, and seafood. In this method, only TCDCA interferes chromatographically with PFOS. In the case where both PFOS and TCDCA (499→124) were detected, an additional SPE clean-up step was performed. Briefly, a 250 mg/6 mL ENVI-Carb cartridge (SupelClean, ENVI-Carb, Millipore Sigma, St. Louis, MO) is conditioned with 4 mL of methanol, then 3 mL of QuEChERS extract is passed through the cartridge. The eluent is then blown to near dryness and reconstituted with 3 mL of methanol before the internal standard is added. The extract is used to analyze PFOS only.

### LC–MS/MS instrumental analysis

A Nexera X2 (Shimadzu, Kyoto, Japan) liquid chromatography system was coupled to a Sciex 6500 plus QTRAP hybrid triple quadrupole/linear ion trap mass spectrometer with an electrospray ion source (ABSciex, Toronto, ON Canada). Analytes were separated using an XBridge BEH C18 analytical column (130 Å, 3.5 µm, 2.1 × 150 mm) with an ACQUITY BEH C18 VanGuard pre-column (130 Å, 1.7 µm, 2.1 × 5 mm) as the guard column (Waters, Milford, MA) and an XBridge BEH C18 column (130 Å, 3.5 µm, 2.1 × 50 mm) as the delay column. The mobile phase consisted of 5 mM ammonium acetate and 5 mM 1-MP in water (A) and methanol (B) and the gradient and other instrumental conditions have been previously reported.[6] The improvements observed with the use of 1-MP in the mobile phase include reduced background and enhanced ionization in a negative mode which results in, on average, 2 × lower method detection limits than without the use of 1-MP.

### LC-HRMS instrumental analysis

Due to matrix interferences and the potential for false positives for PFBA and PFPeA given that they only have one MS/MS transition, liquid chromatography/high-resolution mass spectrometry (LC-HRMS) was used to confirm the presence and concentration of these analytes via accurate mass. The LC-HRMS instrument included a Nexera ultra-performance LC (Shimadzu, Kyoto, Japan) coupled to a Q-Exactive Orbitrap mass spectrometer (Thermo Fisher Scientific, Waltham, MA). The LC separation was performed using the same conditions as described [5] for the LC–MS/MS analysis, except the equilibration hold was increased to 5 min at 10% B at the end of the method due to differences in void volumes between the two instruments. The mass spectrometer was operated using a negative ion polarity, full scan (100–1200 m*/z*) method, with 70 k resolving power, an AGC target of 1e6, and a maximum injection time of 250 ms. The heated electrospray ionization (HESI) source parameters were tuned to minimize in-source fragmentation of PFBA and PFPeA and used a sheath gas flow rate of 35 au, auxiliary gas flow rate of 10 au, a spray voltage of − 2.5 kV, capillary temperature of 350 °C, S-lens RF level of 25, and an auxiliary gas heater temperature of 310 °C. Extracted ion chromatograms were generated for the exact mass of PFBA (*m/z* 212.9792) and PFPeA (*m/z* 262.9760) with a ± 5 ppm mass accuracy tolerance, and the peak intensity values were used for quantification. Concentrations were calculated following the same isotope dilution procedure used for the LC–MS/MS data.

## Results

### Method extension and validation

**Fig. 1:**
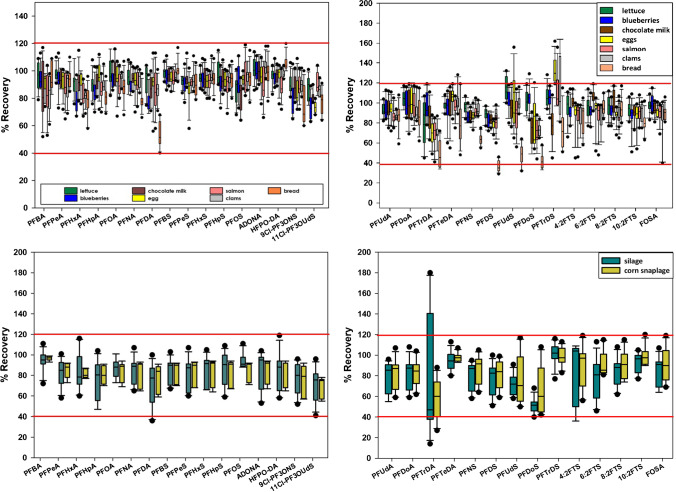
Box plots of 12 total spikes with 3 at each of the following concentrations (0.15 µg/kg, 1 µg/kg, 5 µg/kg, and 15 µg/kg) from the LC–MS/MS analysis. On the left side are box plots of the original 16 analytes and on the right side are the additional 14 analytes. Red lines indicate the 40–120% required recovery range for samples spiked at 1 µg/kg

For the analyte and matrix extension, seven food matrices were chosen to cover samples analyzed in past validations (lettuce, milk (chocolate), salmon, bread) [5, 6], high-priority foods (clams), and matrices with known interferences (eggs, chocolate milk) [6]. For feed samples, silage and corn were chosen due to experience with them being challenging feed matrices with interferences and matrix effects. Each food type was spiked in triplicate at four different concentrations (0.15 µg/kg, 1 µg/kg, 5 µg/kg, and 15 µg/kg), and method detection limits were spiked at 0.05 µg/kg. All spikes were used for validating the LC–MS/MS method for 28 analytes and the three highest spikes were used for validating the LC-HRMS method for PFBA and PFPeA. Percent recoveries are required to fall within the range of 40–120% for a method level of 1 ppb and 60–115% for a method level of 10 ppb and have %RSDs ≤ 22% [13]. In Fig. 1, box plots were created by combining percent recoveries for the triplicate spikes at four concentrations (0.15 µg/kg, 1 µg/kg, 5 µg/kg, 15 µg/kg) for each food type. For the 16 PFAS analytes that have been previously validated, all the recoveries were within acceptable ranges. In past validations, 11Cl-PF3OUdS had recoveries below 40% in bread [5, 6]. MPFHxS is currently the surrogate standard used for quantifying 11Cl-PF3OUdS. If MPFDoA, which is the surrogate with the closest retention time, is used instead of MPFHxS, the recoveries all pass validation. This surrogate was not the optimal surrogate for 11Cl-PF3OUdS in all food matrices so it was only adjusted in the method for 11Cl-PF3OUdS in bread. For the 14 new analytes tested in this extension, some of the long-chain perfluorosulfonic acids (PFSAs) initially did not meet the required minimum 40% recovery in bread (PFDS, PFUdS, PFDoS, PFTrDS), eggs (PFDoS, PFTrDS), chocolate milk, salmon, and clams (PFTrDS) when M8PFOS was used as the surrogate standard for these analytes. Improved recoveries were found when MPFUdA was used for PFUdS, MPFDoA for PFDoS, and MPFTeDA for PFTrDS. With the new surrogates, some of the recoveries still did not pass validation because they were above 120% for PFTrDS in eggs, and for the low spike of PFTrDS for salmon and clams. The recoveries in bread were still less than 40% for some of the spikes of PFDS, PFUdS, and PFDoS. The same improvement was observed in corn and silage where all the recoveries fell within acceptable ranges except those for PFDoS and PFTrDS (with M8PFOS as the surrogate) but then passed when MPFDoA and MPFTeDA were used as matched surrogate standards. Properly matched surrogate standards are more important in complex foods such as bread and silage and this analysis would benefit from the commercial availability of additional long-chain labeled PFSAs for the most accurate quantification. For PFBA and PFPeA, the 1, 5, and 15 µg/kg spikes were run for validation on the HR-MS instrument for all matrices. This instrument is used to verify any positive detect of PFBA or PFPeA based on accurate mass (within ± 5 ppm) and all recoveries fell within acceptable ranges and had %RSDs ≤ 22%, which is required by the guidance document [13] (Fig. 1).

### Comparison to EURL POP LOQs

In August of 2022, the European Union issued a commission recommendation (EU) 2022/1431 for monitoring the presence of PFAS in foods in member states from 2022 to 2025. Then, in December 2022 in commission regulation (EU) 2022/2388, maximum levels were established for four PFAS with European Food Safety Authority (EFSA) establishing tolerable weekly intake values (PFOS, PFOA, PFNA, PFHxS) [14] in specific foods. Required LOQs were given for PFOS, PFOA, PFNA, and PFHxS in food groups where maximum levels have been established (fish meat, eggs, crustaceans, molluscs) [15, 16]. For foods where maximum levels have not been established (milk, fruits, vegetables, fungi, and baby food), both targeted and required LOQs were given for monitoring purposes in the EURL POPs document [16]. In EU 2022/1431, the required LOQs listed in the EURL POPs document were defined as indicative levels and targeted LOQs were the same in both documents for these foods [15, 16]. The approach used to calculate LOQs in the EURL POPs document is defined as the lowest successfully validated concentration which meets the following parameters: ion ratio ± 30%, signal: noise S:N ≥ 3, precision ≤ 20%, trueness between − 20% and + 20% for compliance samples [16].

For foods (salmon, clams, eggs) tested in the validation with current maximum levels established by EU 2022/2388, the four PFAS analytes (PFOS, PFOA, PFNA, PFHxS) all had LOQs below the required LOQs set by the EURL POPs (Table 1). When looking at the required LOQs by the EURL POPs for validated foods for monitoring purposes (blueberries, lettuce, milk), the LOQs calculated using the FDA method were above the required LOQs except for PFNA and PFHxS in milk. In order to make a more accurate comparison to the EU-required LOQs, spikes of 5, 10, and 20 ng/kg were made in triplicate to determine the lowest successfully validated level using the FDA method. At 20 ng/kg, all of the data was able to meet the required criteria (80–120% recovery, ≤ 20% RSD) for milk, lettuce, and blueberries. In an attempt to adjust the method to decrease the LOQ for these matrices, an additional concentration step was added for the produce and milk samples. After the dSPE step, 5 mL (instead of 1 mL) of the extract was concentrated to 1 mL and used for SPE analysis and the final extract was reduced to 0.5 mL instead of 1 mL. This extra step allowed the LOQs to pass a 5 ng/kg validated level for blueberries and lettuce and a 10 ng/kg validated level for milk. In addition to the required LOQs for monitoring purposes which are also described as indicative levels in EU 2022/1431, lower targeted LOQs are also described by the EURL POPs for foods without maximum limits. Currently, to reach these lower targeted LOQs using the FDA method, adjustments in analytical instrumentation would need to be made.

**Table 1:** EURL POPs required limits of quantification (µg/kg) compared to FDA LOQs

	PFOS	PFOA	PFNA	PFHxS
**EURL POPs—fruits, vegetables required LOQ for monitoring**	**0.010**	**0.010**	**0.005**	**0.015**
FDA method lettuce LOQ	0.050	0.050	0.050	0.020
FDA method blueberries LOQ	0.050	0.030	0.050	0.020
FDA method lettuce and blueberries LOQ calculated using EURL POPs guidance	0.020	0.020	0.020	0.020
FDA method lettuce and blueberries LOQ with concentration step calculated using EURL POPs guidance	*0.005*	*0.005*	*0.005*	*0.005*
**EURL POPs—milk required LOQ for monitoring**	**0.020**	**0.010**	**0.050**	**0.060**
FDA chocolate milk LOQ	0.030	0.090	*0.050*	*0.010*
FDA method chocolate milk LOQ calculated using EURL POPs guidance	*0.020*	0.020	*0.020*	*0.020*
FDA method chocolate milk LOQ with concentration step calculated using EURL POPs guidance	*0.010*	*0.010*	*0.010*	*0.010*
**EURL fish meat and meat of terrestrial animals required LOQ for compliance**	**0.100**	**0.100**	**0.100**	**0.100**
FDA salmon LOQ	*0.060*	*0.050*	*0.090*	*0.050*
**EURL eggs, crustaceans, and molluscs required LOQ for compliance**	**0.300**	**0.300**	**0.300**	**0.300**
FDA eggs LOQ	*0.060*	*0.160*	*0.040*	*0.040*
FDA clams LOQ	*0.050*	*0.180*	*0.090*	*0.030*

*FDA LOQ is at or below LOQ required by EURL POPs*

#### MDLs

The FDA currently uses MDLs as defined in 40 CFR 136 Appendix B as the lowest concentration reportable for screening purposes for dietary exposure assessments. The MDL is equal to the sample standard deviation of low-level spiked matrix (0.05 µg/kg) multiplied by the corresponding student’s *t*-value for a single-tailed 99th percentile *t*-statistic with *n*-1 degrees of freedom [14, 17]. This results in the minimum concentration that is significantly different from zero and varies for different analytes and matrices. For determining the LOQ, the sample standard deviation is multiplied by 10.

It is recognized that the MDL and LOQ represent different concepts where the MDL defines the value which can be detected and the LOQ the value which can be quantified. Since the FDA is currently reporting out all data above the MDL, the MDLs for lettuce, blueberry, and milk were also compared to the LOQs required for monitoring purposes to compare differences in concentrations reported in monitoring the food supply in Europe and the US. When comparing the FDA MDL to the EURL LOQ for blueberries, PFOA and PFHxS were below and PFOS was 0.015 µg/kg compared to the required 0.010 µg/kg. For lettuce, PFHxS was below and PFOS and PFOA were both 0.014 µg/kg compared to the required 0.010 µg/kg. PFNA was higher (0.014 and 0.013 µg/kg compared to the required 0.005 µg/kg). For the MDL milk comparison, 3 out of 4 analytes were below the required LOQs by the EURL POPs and PFOA was 0.020 µg/g compared to the required 0.010 µg/kg.

## Discussion

The expansion of the FDA’s analytical method for PFAS in foods from 16 to 30 analytes allows for the determination of additional analytes that have the potential to be found in the US food supply. This expanded method will allow these analytes to be determined in future Total Diet Study samples, which are used for assessing dietary exposure, and in other studies involving foods affected by environmental contamination. The FDA method can quantify analytes with maximum levels identified in Commission Regulation (EU) 2022/2388 in priority foods below the recommended LOQs. For monitoring purposes, the FDA reports values above the MDLs and these are comparable with LOQs required by the EURL POPs for surveillance samples. This indicates that values above similar thresholds are being reported in the US and Europe but an extra concentration step is needed in the FDA method to reach the required LOQs for produce and milk. For exposure assessments, the lowest possible LOQs are desired and continued improvements in instrumentation and methods are needed to reach EURL POPs targeted LOQs. By continuing to expand and improve the analytical method for PFAS in foods, the FDA can improve its knowledge of PFAS dietary exposure and surveillance of PFAS of foods in the US marketplace.
